# Propagation of Human Prostate Cancer Stem-Like Cells Occurs through EGFR-Mediated ERK Activation

**DOI:** 10.1371/journal.pone.0061716

**Published:** 2013-04-19

**Authors:** Adrian P. Rybak, Alistair J. Ingram, Damu Tang

**Affiliations:** 1 Division of Nephrology, Department of Medicine, McMaster University, Hamilton, Ontario, Canada; 2 Father Sean O’Sullivan Research Institute, Hamilton, Ontario, Canada; 3 The Hamilton Centre for Kidney Research (HCKR), St. Joseph’s Hospital, Hamilton, Ontario, Canada; The University of Texas M.D Anderson Cancer Center, United States of America

## Abstract

Prostate cancer stem-like cells (PCSCs) are being intensely investigated largely owing to their contributions towards prostate tumorigenesis, however, our understanding of PCSC biology, including their critical pathways, remains incompletely understood. While epidermal growth factor (EGF) is widely used in maintaining PCSC cells in vitro, the importance of EGF-dependent signaling and its downstream pathways in PCSC self-renewal are not well characterized. By investigating DU145 sphere cells, a population of prostate cancer cells with stem-like properties, we report here that epidermal growth factor receptor (EGFR) signaling plays a critical role in the propagation of DU145 PCSCs. Activation of EGFR signaling via addition of EGF and ectopic expression of a constitutively-active EGFR mutant (EGFRvIII) increased sphere formation. Conversely, inhibition of EGFR signaling by using EGFR inhibitors (AG1478 and PD168393) and knockdown of EGFR significantly inhibited PCSC self-renewal. Consistent with the MEK-ERK pathway being a major target of EGFR signaling, activation of the MEK-ERK pathway contributed to EGFR-facilitated PCSC propagation. Modulation of EGFR signaling affected extracellular signal-related kinase (ERK) activation. Inhibition of ERK activation through multiple approaches, including treatment with the MEK inhibitor U0126, ectopic expression of dominant-negative MEK1(K97M), and knockdown of either ERK1 or ERK2 resulted in a robust reduction in PCSC propagation. Collectively, the present study provides evidence that EGFR signaling promotes PCSC self-renewal, in part, by activating the MEK-ERK pathway.

## Introduction

Prostate cancer is the most common male malignancy and the second leading cause of cancer-related deaths in males in Western countries [1], [2]. During the process of prostate tumorigenesis, oncogenic signaling pathways promote the progression of hormone-dependent carcinomas to hormone refractory prostate cancer (HRPC), the major contributing factor in prostate cancer fatalities [3], [4]. Although the exact mechanisms responsible for the initiation and progression of prostate cancer remain largely unknown, prostate cancer stem-like cells (PCSCs) are widely regarded as being critical in prostate tumorigenesis and its development towards HRPC disease [5]–[7].

Despite the mounting evidence suggesting the existence of PCSCs, identification of human PCSCs in vivo has appeared to be a challenging task. This challenge is largely due to the heterogeneous nature of prostate cancer and the limited samples available from clinical sources. Our limited understanding of PCSCs has also contributed to the inability to isolate and propagate PCSCs from human primary carcinomas. To advance our knowledge of PCSCs, several research groups, including ours, have enriched for PCSCs from human prostate cancer cell lines. This is largely attributable to the demonstration that cancer stem cells (CSCs) can be studied using the sphere culture assay under serum-free (SF) media conditions. This assay has been used to derive and propagate CSCs from brain [8], breast [9], colon [10] and prostate cancer cells [11]–[16] in vitro. More importantly, the sphere culture approach has allowed the propagation of prostate cancer stem-like cells that display CSC properties of self-renewal and the ability to initiate tumor formation in vivo [11], [12], [15], [17].

Sphere culture commonly involves propagating stem-like cells in SF media supplemented with epidermal growth factor (EGF) and basic fibroblast growth factor (bFGF) [8]–[13]. Although the presence of both EGF and bFGF allows the generation of spheres from DU145 cells [11], [12], [17], whether this is the ideal condition for sphere generation and PCSC maintenance for a prolonged period of time remains unclear. In our recent investigation, we have shown that EGF plays a critical role in long-term propagation of DU145 PCSCs, and that these stem-like cells were capable of initiating tumors with a significantly enhanced ability in non-obese, diabetic/severe combined immunodeficient (NOD/SCID) mice [11]. However, the role of EGFR signaling, along with its downstream pathways that are required for DU145 PCSC propagation remain to be characterized.

In our effort to advance this knowledge, we demonstrate here that the EGFR-ERK connection plays an important role in the propagation of DU145 PCSCs. Although these PCSCs are able to propagate in the absence of exogenous EGF, activation of EGFR signaling is critical for the maintenance of DU145 PCSCs as experimental manipulation of EGFR signaling affected DU145 PCSC propagation. Additionally, modulation of EGFR signaling in DU145 PCSCs profoundly affected ERK activation. Furthermore, inhibition of ERK activation through the use of a MEK inhibitor, ectopic expression of dominant-negative MEK1(K97M), and knockdown of endogenous ERK1 or ERK2, collectively reduced the propagation of DU145 PCSCs. Taken together, these results highlight a contribution of MEK-ERK signaling for EGFR-mediated PCSCs self-renewal.

## Materials and Methods

### Cell Culture and Propagation of DU145 PCSCs

DU145 human prostate cancer cells and 293T human embryonic kidney cells were obtained from American Type Culture Collection (ATCC, Manassas, VA, USA), and were cultured according to ATCC instructions.

DU145 human prostate cancer stem-like cells were isolated and propagated as previously published [11]. Briefly, DU145 monolayer cells were enzymatically-dissociated into single cells using phenol red-free TrypLE Express solution (Life Technologies, Calsbad, CA, USA) and 40 µm cell strainers (BD Biosciences, Franklin Lakes, NJ, USA), and subsequently resuspended at a sub-clonal (5 cells/µl) density in serum-free (SF) media (DMEM/F12 at a 3∶1 mixture) (Life Technologies) containing 0.4% bovine serum albumin (BSA) (Bioshop Canada Inc., Burlington, ON, Canada) and 0.2× B27 lacking Vitamin A (Life Technologies) in T75 flasks (15 ml total volume) designated for suspension cell culture (Sarstedt Inc., Newton, NC, USA). When examining the effect of EGF treatment on sphere formation, recombinant EGF (Sigma-Aldrich, St. Louis, MO, USA) was added at a concentration of 10 or 20 ng/ml. Typical spheres formed in 10 to 12 days. Sphere cells were sub-cultured by enzymatic dissociation, strained, and then resuspended in the above medium at sub-clonal density.

### Sphere Formation Assays

To evaluate the formation of primary spheres, DU145 cells from sub-confluent (∼80%) monolayer cultures were resuspended in the above SF media at a density of 2.5×10^3^ cells into individual wells of a 24-well culture plate (3 wells per treatment) (Corning, Corning, NY, USA). Cells were seeded in a volume of 0.5 mL/well, unlike 1 ml/well as previously published [11]. The spheres that formed after 12 days of culture were counted. To measure the sphere-forming capacity of secondary or established spheres, spheres cells were individualized by enzymatic dissociation, strained, and then plated at a density of 5×10^2^ or 1×10^3^ cells/well in 24-well plates (3 wells per treatment), unless otherwise specified. The spheres that formed were counted after 12 days of culture.

### Plasmids and Virus Production

EGFRvIII/pLNCX was kindly provided by Dr. Khalid Al-Nedawi (Hamilton Centre for Kidney Research, Hamilton, ON, Canada). EGFR(K721A)/pLHCX was kindly provided by Dr. Joan Krepinsky (Hamilton Centre for Kidney Research). Construction of MEK1(K97M)/pLHCX has been previously described [18]. Short-hairpin ERK1 and ERK2 targeting (shERK1 and shERK2, respectively) vectors were constructed by annealing and subcloning the previously published targeting sequences (ERK1: GCCATGAGAGATGTCTACA; ERK2: GAGGATTGAAGTAGAACAG) [19] as a hairpin into the pRIH retroviral vector, according to our published conditions [18]. The short-hairpin control (shLacZ) vector contains a sequence targeting *Escherichia coli* β-galactosidase (shLacZ target sequence: GCAGTTATCTGGAAGATCA). High titres of empty vector (EV)/pLNCX, EGFRvIII/pLNCX, EV/pLHCX, EGFR(K721A)/pLHCX, MEK1(K97M)/pLHCX, shLacZ/pRIH, shERK1/pRIH and shERK2/pRIH retrovirus were produced using 293T cells, as previously described [18]. DU145 cells were subsequently infected with the specific retrovirus, and infected cells were selected in hygromycin (0.5 mg/ml; Life Technologies) for pLHCX and pRIH-based infections, or G418 (1 mg/ml; Sigma-Aldrich) for pLNCX-based infections. Ectopic EGFRvIII and MEK1(K97M) expression, or knockdown of endogenous ERK1 or ERK2, were verified by Western blot analysis.

Short-hairpin EGFR targeting lentiviral vectors were purchased from Sigma-Aldrich, with the targeting sequences (shEGFR1: GCTGCTCTGAAATCTCCTTTA; shEGFR2: GCCACAAAGCAGTGAATTTAT) cloned as a hairpin in the pLKO.1 vector, while a non-specific shRNA (plasmid 1864; Addgene, Cambridge, MA, USA) was used as a control (shCTRL). Production of shEGFR and shCTRL lentivirus was carried out by co-transfecting each shRNA plasmid (10 µg) with plasmids (10 µg each) necessary for third-generation lentiviral production (pRSV-REV, pCMV-VSV-G, pMDLg/pRRE) [20], kindly provided by Dr. Bryan E. Strauss (University of São Paulo School of Medicine, São Paulo, Brazil), into 293T cells using the calcium phosphate method. Sixty hours (h) post-transfection, the supernatants containing VSV-G pseudotyped, replication-incompetent lentiviral particles were collected, filtered through a 0.45 µm filter and centrifuged for 2 h at 48,000 g. Viral pellets were resuspended in 1 ml of media containing 10 µg/ml polybrene (Sigma-Aldrich) and added to monolayer cells for 2 h in a humidified 37°C incubator, with periodic mixing at 20 min intervals, to allow for cell infection. Infection was selected with puromycin (1 µg/ml; Sigma-Aldrich).

For infection of sphere cells, the shEGFR2 and shCTRL lentivirus pellets were resuspended in 2 ml of serum-free media containing 0.4% BSA, 0.2× B27 (lacking Vitamin A) and 10 µg/ml polybrene and added to individualized (3×10^5^) cells for 2 h in a humidified 37°C incubator, with periodic mixing at 20 min intervals, to allow for cell infection. Sphere cells were subsequently seeded at a density of 10^4^ cells/ml in T75 flasks designated for suspension cell culture (Sarstedt Inc.). Puromycin (1 µg/ml) was added to the sphere cultures at 48 hours post-infection.

### Anchorage-Independent Growth Assay

DU145 sphere cell cultures were plated in individual wells of six-well plates at a density of 10^4^ cells/well as previously described [11]. After 8 weeks, phase contrast images were taken in 5 random fields per well using the Zeiss Axiovert 200 M microscope (AxioVision 3.1 software) at 25X magnification, and the colonies consisting of ≥50 cells were counted. Phase contrast images were subsequently processed using CorelDRAW Graphics Suite X4 software (Corel, Ottawa, ON, Canada).

### EGFR and MEK Inhibition Studies

The EGFR inhibitors AG1478 and PD168393 (Calbiochem, Darmstadt, Hesse, Germany) and the MEK inhibitor U0126 (Promega, Madison, WI, USA) were dissolved in dimethylsulfoxide (DMSO; Sigma-Aldrich) and used at the concentrations indicated. DMSO was administered at an identical volume as a control (mock treatment). EGFR inhibitor (AG1478 or PD168393), MEK (U0126) or DMSO (mock) treatment was administered at the time of seeding sphere cells. Sphere formation was quantitated as previously indicated. Inhibition of EGFR and MEK1 kinase activity (as determined by EGFR and ERK1/2 phosphorylation status, respectively) was verified by Western blot analysis.

Antibody-based EGFR function blocking experiments on sphere cells were carried out as previously described [21]. Briefly, azide-free EGFR blocking mouse monoclonal (Ab-1, clone 528) antibody (Thermo Fisher Scientific, Waltham, MA, USA) or mouse IgG2a isotype control antibody (R&D Systems, Minneapolis, MN, USA) were added to sphere cells (at the time of seeding) at a concentration of 4 µg/ml in the presence or absence of recombinant EGF (10 ng/ml). Spheres were allowed to form and were quantitated as previously indicated.

### Western Blot Analysis

Whole cell lysates were prepared in a lysis buffer containing 20 mM Tris (pH 7.4), 150 mM NaCl, 1 mM EDTA, 1 mM EGTA, 1% Triton X-100, 25 mM sodium pyrophosphate, 1 mM NaF, 1 mM β-glycerophosphate, 0.1 mM sodium orthovanadate, 1 mM PMSF, 2 µg/ml leupeptin and 10 µg/ml aprotinin. For each sample, a total of 50 µg of whole cell lysate was used unless otherwise specified. Resolved lysates (10% SDS-polyacrylamide gels) were transferred onto Amersham Hybond-ECL membranes (GE Healthcare, Little Chalfont, Buckinghamshire, United Kingdom). The membranes were blocked with 5% w/v non-fat dry skim milk at room temperature for 1 h, and then incubated with the indicated primary antibodies in 10 ml of antibody dilution buffer (1× Tris-buffered saline containing 0.1% Tween-20 (TBST), and 5% BSA) with gentle rocking at 4°C overnight. Primary antibodies used for probing were as follows: rabbit anti-human phospho-EGFR (Tyr1068) (Sigma-Aldrich, E6154, 1∶1000), rabbit anti-human EGFR (#4267, 1∶1000), rabbit anti-human phospho-AKT (Ser473) (#4058, 1∶1000), rabbit anti-human phospho-ERK1/2 (Thr202/Tyr204) (#9101, 1∶1000), rabbit anti-human ERK1/2 (#9102, 1∶1000), rabbit anti-human phospho-STAT3 (Tyr705) (#9145, 1∶2000), mouse anti-human STAT3 (#9139, 1∶1000) from Cell Signaling Technology (Danvers, MA, USA), goat anti-human AKT (C-20, 1∶1000), mouse anti-human MEK1 (H-8, 1∶1000) and mouse anti-human α-tubulin (TU-02, 1∶500) from Santa Cruz Biotechnology (Santa Cruz, CA, USA). Membranes were subsequently washed with 1× TBST solution and incubated (1 h at room temperature) with either donkey anti-goat-IgG-horse radish peroxidase (HRP) (Santa Cruz Biotechnology, sc-2033, 1∶3000), donkey anti-mouse-IgG-HRP (GE Healthcare, NA931V, 1∶3000) or donkey anti-rabbit-IgG-HRP (GE Healthcare, NA934V, 1∶3000) conjugated secondary antibodies. Immunoblot signals were detected using Amersham ECL Western blotting detection reagents (GE Healthcare) and then exposed to x-ray film (Thermo Fisher Scientific). The quantification of immunoblot band densities was determined using ImageJ software (version 1.43u; W. Rasband, National Institute of Health).

### Statistical Analysis

All quantitative data are presented as mean ± S.E.M. and comparisons were made using two-tailed independent Student’s *t*-tests. A *p*-value <0.05 was considered statistically significant.

## Results

### DU145 Spheres Propagated in the Absence of Exogenous Mitogens Display EGFR Signal Activation

**Figure 1:**
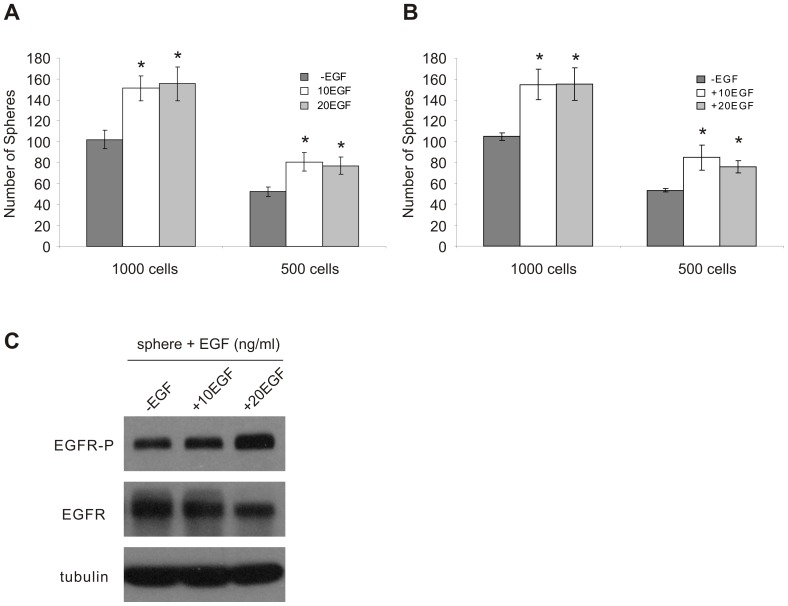
Formation and maintenance of DU145 spheres occurs independently of exogenous mitogens, with EGF treatment enhancing sphere formation. A) EGF enhances sphere formation independent of the cell seeding density. DU145 spheres were generated and maintained in serum-free media containing 0.4% BSA and 0.2× B27 (SF), and supplemented with or without EGF (10 or 20 ng/ml; 10EGF or 20EGF, respectively) for three passages to establish EGF-free (-EGF) and EGF-stimulated sphere cultures prior to experimentation. Sphere cells were seeded at a density of 5×10^2^ and 1×10^3^ cells/well (1×10^3^ and 2×10^3^ cells/ml, respectively) in 24-well plates (three replicates per treatment). Six experiments were conducted. Spheres maintained in EGF-supplemented SF media display enhanced sphere numbers compared to -EGF sphere cultures. Spheres maintained in SF media containing >10 ng/ml EGF do not display additional increases in sphere-forming capacity. B) Treatment of -EGF spheres with increasing concentrations of EGF (+10EGF or +20EGF, respectively) increased sphere formation, and C) enhanced activation of EGFR signaling (EGFR phosphorylation at Tyr1068; EGFR-P). Sphere numbers are displayed as mean ± S.E.M. of four independent experiments (*p<0.05 in comparison to the respective -EGF media condition; two-tailed independent Student’s *t*-test).

We have previously reported that while EGF supplementation significantly promoted the generation and propagation of DU145 spheres, the formation of spheres did not require the addition of exogenous EGF [11] suggesting that EGFR signaling may be activated in the absence of exogenous EGF. To investigate this possibility, we have generated spheres from DU145 cells in the absence (-EGF) and presence of EGF (10 or 20 ng/ml EGF; 10EGF or 20EGF, respectively) and subsequently propagated these established spheres for three passages, under the identical conditions in which primary spheres were generated, prior to evaluating their sphere-forming capacity. Spheres generated in the -EGF media condition can be maintained in a consistent manner for at least three passages. Furthermore, the ability to generate and maintain spheres in the -EGF condition (Figure 1A) suggests that propagation under EGF-free (also without the addition of other external growth factors) media conditions is an intrinsic property of DU145 PCSCs. This possibility is supported by the fact that the number of spheres maintained after seeding 5×10^2^ cells/well was approximately half of that propagated after seeding 1×10^3^ cells/well (Figure 1A). Furthermore, this possibility is also in line with our recent observation that -EGF spheres have been propagated in -EGF media for more than 15 consecutive passages, in the consistent manner as indicated in Figure 1A, by the time this manuscript was prepared (data not shown). Although EGF enhanced sphere propagation compared to sphere cells grown in -EGF media (10EGF and 20EGF compared to -EGF), 20EGF did not further stimulate sphere number in comparison to the 10EGF treatment. This suggests that an EGF-induced threshold exists (Figure 1A).

To examine whether spheres generated and maintained in the -EGF media condition respond to EGFR signal activation, we have cultured established, EGF-free spheres in the presence of exogenous EGF. Not only did these EGF-free sphere cells respond to exogenous EGF treatment when seeded at sub-clonal densities of 5×10^2^ and 1×10^3^ cells/well (1×10^3^ and 2×10^3^ cells/ml, respectively) (Figure 1B), but they also respond to EGF to the same level as those spheres generated and maintained in EGF-containing media (comparing Figure 1B with Figure 1A). These observations indicate that in the absence of exogenous EGF, spheres may be able to activate EGFR signaling. In support of this possibility, phosphorylation of EGFR at tyrosine (Y) 1068 (EGFR-P), a commonly used surrogate marker of EGFR signal activation [22], was readily detected in -EGF and +EGF (10EGF and 20EGF) sphere cells (Figure 1C). Taken together, the above observations support the notion that EGFR signaling plays an important role in the propagation of DU145 PCSCs.

### Enforced EGFR Signal Activation Renders DU145 PCSCs Non-responsive to EGF Treatment

**Figure 2:**
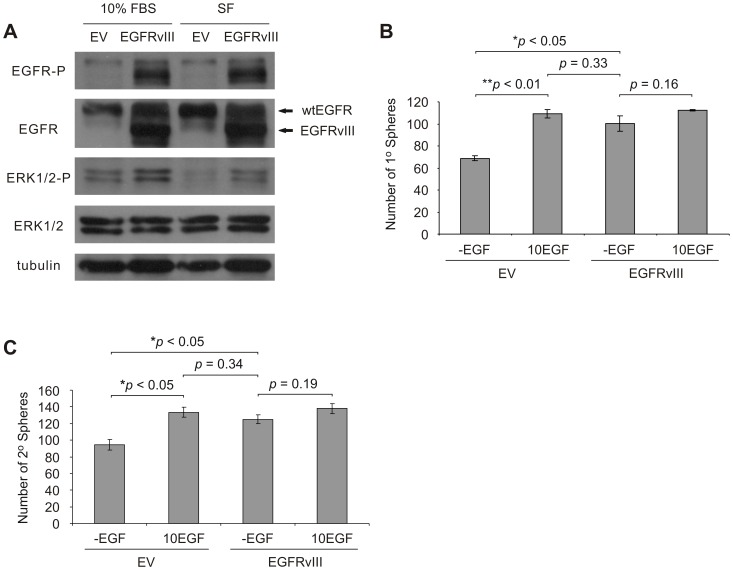
Expression of constitutively-active EGFRvIII enhances DU145 sphere formation and maintenance. A) Western blot analysis of EGFRvIII expression and activation (Tyr1068 phosphorylation; EGFR-P) in DU145 parent (monolayer) cells grown in serum-supplemented (10% FBS) and serum-free (SF) media. EGFRvIII expression in DU145 cells activates ERK signaling. ERK activation was determined by examining the phosphorylation of ERK1 and ERK2 proteins at Thr202 and Tyr204 residues, respectively (ERK1/2-P). B) Primary (1°) and C) secondary (2°) sphere formation following EGFRvIII expression in DU145 cells compared to empty vector (EV) control cells. Cells were seeded in serum-free media containing 0.4% BSA, 0.2× B27 and lacking exogenous growth factors (-EGF), or with EGF supplemented (10 ng/ml; 10EGF), at a density of 2.5×10^3^ (1° sphere formation) or 1×10^3^ cells/well (2° sphere formation) in 24-well plates (0.5 ml/well; three replicates per treatment). Experiments were conducted in triplicate. Sphere numbers are displayed as mean ± S.E.M. (the *p* values for the indicated comparisons were obtained by two-tailed independent Student’s *t*-test).

To further ascertain the role of EGFR signal activation in DU145 PCSCs, the constitutively-active EGFR mutant, EGFR variant III (EGFRvIII) [23], was overexpressed in DU145 monolayer cells (Figure 2A). EGFRvIII-expressing cells displayed elevated levels of Tyr1068 phosphorylation (EGFR-P) and ERK activation (Thr202/Tyr204 phosphorylation on ERK1/2; ERK1/2-P) in SF media (Figure 2A), demonstrating that ectopic EGFRvIII expression resulted in constitutive EGFR signaling. In comparison to empty vector (EV) transduced DU145 cells, DU145 EGFRvIII-expressing cells generated more primary spheres in -EGF media (Figure 2B). Furthermore, EGF supplementation promoted the generation of primary spheres from DU145 EV cells but not EGFRvIII-expressing cells (Figure 2B). However, EGFRvIII-expressing sphere cells can form primary spheres in -EGF media at a similar level as DU145 EV sphere cells propagated in the presence of exogenous EGF (Figure 2B). Therefore, DU145 EGFRvIII-expressing cells possess a comparable capacity for primary sphere generation as DU145 EV cells stimulated with recombinant EGF (Figure 2B). Similar observations were also obtained in sphere propagation (i.e. the production of secondary spheres upon seeding 10^3^ cells/well) (Figure 2C). Collectively, these results support our hypothesis that EGFR signaling contributes to the propagation of DU145 PCSCs.

### EGFR Blockade Reduces the Self-renewal Capacity of DU145 PCSCs

**Figure 3:**
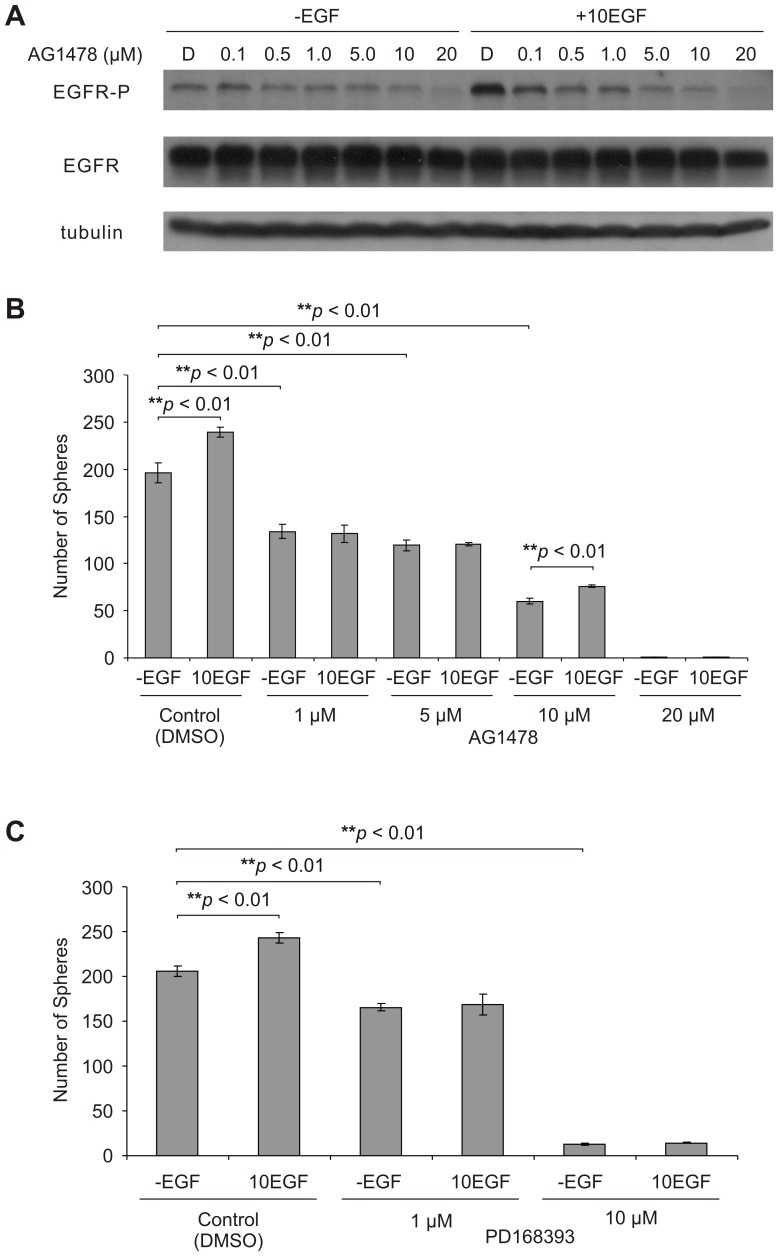
EGFR signaling blockage reduces the self-renewal activity of DU145 PCSCs. A) Western blot analysis of whole cell lysates following 24 hour treatment of DU145 spheres with DMSO (D) or increasing concentrations of AG1478 (0.1–20 µM concentration range), in the presence or absence of exogenous EGF (10 ng/ml; 10EGF). Sphere formation of EGF-free sphere cells was inhibited following treatment with EGFR inhibitors B) AG1478 or C) PD168393 at various doses in the presence (10EGF) or absence (-EGF) of exogenous EGF (10 ng/ml). Sphere cells were seeded at a density of 2×10^3^ cells/well (0.5 ml/well; three replicates per treatment). EGFR inhibitor (AG1478 or PD168393) or DMSO (mock) treatment was administered at the time of seeding. Spheres that formed were counted 12 days post-seeding. Sphere numbers are displayed as mean ± S.E.M. (the *p* values for the indicated comparisons were obtained by two-tailed independent Student’s *t*-test).

To further investigate the requirement of EGFR signaling in the propagation (self-renewal) of DU145 PCSCs, we examined the effect of EGFR signal inhibition on sphere maintenance. Sphere cells were treated with increasing concentrations of AG1478 (Tyrphostin), a selective inhibitor of EGFR (ErbB1) kinase activity that functions by sequestering it in its inactive form [24]. AG1478 treatment was examined at 24 h post-treatment, as EGF treatment achieved the highest levels in EGFR-P in sphere cells at this time point (Figure S1). AG1478 treatment reduced EGFR activation in a dose-dependent fashion in sphere cells, with and without the addition of exogenous EGF (Figure 3A). Furthermore, AG1478 dose-dependently reduced sphere propagation, as the number of spheres generated from established sphere cell cultures was reduced by AG1478 treatment, irrespective of whether exogenous EGF was present (Figure 3B). Similar observations were also obtained using the EGFR inhibitor PD168393 [25] (Figure 3C). This demonstrates that inhibition of EGFR signaling blocks the self-renewal capacity of DU145 PCSCs.

**Figure 4:**
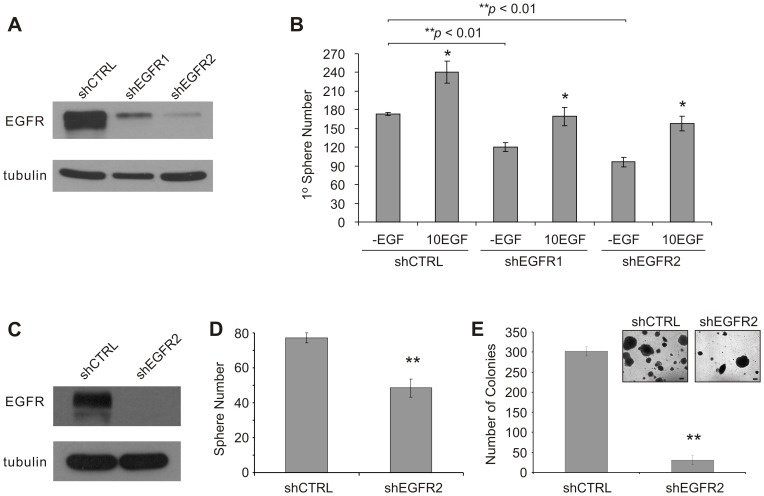
EGFR knockdown reduces the sphere-forming capacity and self-renewal activity of DU145 PCSCs. A) DU145 monolayer cells were infected with EGFR shRNA lentiviral constructs and selected with puromycin in order to generate stable cell lines. Western blot analysis of various EGFR knockdown (shEGFR) cells was carried out to evaluate the EGFR knockdown efficiency relative to the non-specific shRNA control (shCTRL) cell line. B) Stable EGFR knockdown cells form fewer primary (1°) spheres compared to shCTRL cells. Cells were seeded at a density of 2.5×10^3^ cells/well (0.5 ml/well; three replicates per treatment). Sphere numbers are displayed as mean ± S.E.M. of three independent experiments (*p<0.05 in comparison to the respective -EGF media condition for each cell line; two-tailed independent Student’s *t*-test). C) Efficient EGFR knockdown in established DU145 spheres can also be achieved. Cells from EGF-free (-EGF) spheres were infected with shCTRL and shEGFR2 lentivirus. Loss of EGFR protein expression significantly reduces the D) number of DU145 spheres grown under EGF-free media conditions. EGFR knockdown (shEGFR2) and shCTRL sphere cells were seeded at a density of 1×10^3^ cells/well (three replicates per cell line). The number of spheres that formed was counted 12 days post-seeding. Sphere number is displayed as mean ± S.E.M. of three independent experiments (**p<0.01; two-tailed independent Student’s *t*-test). E) EGFR knockdown in DU145 sphere cells significantly reduced their in vitro anchorage-independent growth. The total number of colonies in 5 random fields (at 25X magnification) was counted and represented as mean ± S.E.M. of three independent experiments (**p<0.01; two-tailed independent Student’s *t*-test). Representative phase contrast images of colonies are shown at 50X magnification (inset). Scale bar is equal to 100 µm.

To further address whether loss of EGFR signaling affects the ability of DU145 PCSCs to maintain themselves, DU145 monolayer cells were infected with shRNA constructs targeting EGFR. These constructs efficiently knocked-down EGFR, with slightly variations in the levels of EGFR knockdown within the respective stable cell lines (Figure 4A). The shEGFR construct #2 (shEGFR2) achieved a greater knockdown than shEGFR1 (95% vs 70%, respectively) (Figure 4A). Stable EGFR knockdown cell lines could be maintained in complete media. However, ectopic expression of EGFR(K721A), an EGFR mutant lacking protein tyrosine kinase activity [26], resulted in cell death and prevented the generation of a stable cell line (data not shown). By using these stable EGFR knockdown cell lines, we were able to show that EGFR knockdown in DU145 cells reduced their capacity to generate primary spheres compared to shCTRL cells (Figure 4B). The ability of EGFR knockdown to affect sphere maintenance was also examined. Stable EGFR knockdown in sphere cells (Figure 4C) reduced the number of subsequent spheres formed in the -EGF media condition (Figure 4D).

An important feature of cellular transformation in vitro, which closely correlates with in vivo tumorigenicity in immunocompromised mice [27]–[29], is the ability of tumorigenic cells to propagate under anchorage-independent conditions. To address whether the loss of EGFR expression affects the anchorage-independent growth of DU145 PCSCs, established EGFR knockdown sphere cells were seeded in serum-supplemented, soft agar-containing media. EGFR knockdown in sphere cells resulted in fewer soft agar colonies compared to shCTRL sphere cells under differentiating (ie. serum-supplemented) conditions (Figure 4E). Taken together, we demonstrated that EGFR signaling is critical for maintaining DU145 PCSC propagation, which is consistent with the observed reduction in soft agar colony numbers following EGFR knockdown.

### MEK-ERK Signaling Regulates the Self-renewal Properties of DU145 PCSCs

After the demonstration that EGFR signaling is critical in the generation and maintenance of PCSCs, we further examined the downstream mechanisms responsible for the observed EGFR activity. EGFR signaling is well known to initiate signaling events involving the activation of the PI3K-AKT and Raf-MEK-ERK (mitogen activated protein kinases; MAPK) pathways [30]. In addition, EGFR signaling has been shown to activate signal transducer and activator of transcription proteins (STAT) [31], particularly STAT3-mediated signaling [32]. Activation of STAT3 involves phosphorylation at Tyr705 [33], and STAT3 has been shown to be activated in prostate cancers [34], [35]. Our previous work has suggested that in our system, other downstream signaling proteins, rather than AKT, must be critical for EGF-enhanced DU145 sphere formation [11]. While AKT may contribute to DU145 sphere propagation, we have further expanded on our previous report to examine the contributions of the ERK pathway towards EGFR-dependent propagation of DU145 PCSCs.

**Figure 5:**
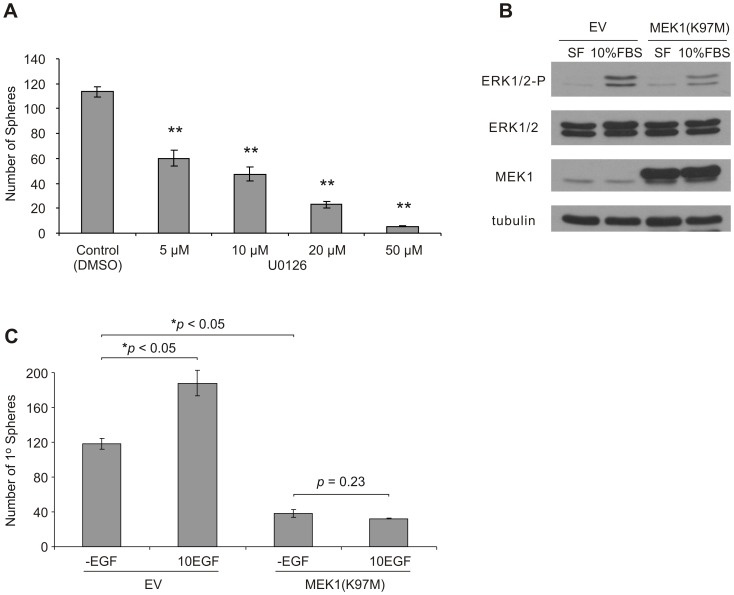
MEK1-dependent signal blockage inhibits DU145 sphere formation. A) Inhibition of ERK signaling following treatment of DU145 sphere cells with increasing concentrations (µM) of U0126 (MEK inhibitor) reduces their capacity to form subsequent spheres. U0126 or DMSO (mock) treatment was administered at the time of seeding. Sphere cells were seeded at a density of 1×10^3^ cells/well (0.5 ml/well; three replicates per treatment). The number of spheres (mean ± S.E.M.) that formed after 12 days of culture was counted (**p<0.01 compared to DMSO control; two-tailed independent Student’s *t*-test). B) Dominant-negative MEK1(K97M) (pLHCX) cells display reduced ERK1/2 activation compared to empty vector (EV; pLHCX) control cells. DU145 EV and MEK1(K97M) cells were serum-starved for 12 h, and subsequently stimulated with serum-supplemented (10% FBS) or serum-free (SF) media for 60 min. C) MEK1(K97M) cells display reduced primary (1^o^) sphere-forming capacity compared to EV cells when seeded in serum-free media lacking EGF (-EGF) or containing 10 ng/ml EGF (10EGF). Cells were seeded at a density of 2.5×10^3^ cells/well (three replicates per treatment). Sphere numbers are displayed as mean ± S.E.M. of three independent experiments (the *p* values for the indicated comparisons were obtained by two-tailed independent Student’s *t*-test).

In this effort, we were able to show that EGF stimulation of EGF-free DU145 sphere cells resulted in ERK activation, but not STAT3 activation, in a time-dependent manner (Figure S1). Furthermore, the MEK inhibitor, U0126 [36], dose-dependently reduced the self-renewal capacity of DU145 PCSCs (Figure 5A). As expected, U0126 inhibited ERK activation in DU145 PCSCs (Figure S2). To consolidate the results implicating MEK-ERK signaling in DU145 PCSC propagation, we have expressed the dominant-negative MEK1(K97M) [37] in DU145 cells (Figure 5B). MEK1(K97M) cells displayed reduced ERK activation compared to empty vector (EV) control cells following FBS-stimulation (10%) of serum-deprived cells (Figure 5B). In comparison to EV cells, MEK1(K97M) cells displayed a significant reduction in generating primary spheres (Figure 5C). In support of MEK-ERK signaling being critical to EGFR-mediated generation of DU145 PCSCs, exogenous EGF was incapable of further enhancing primary sphere formation of MEK1(K97M) cells (Figure 5C).

**Figure 6:**
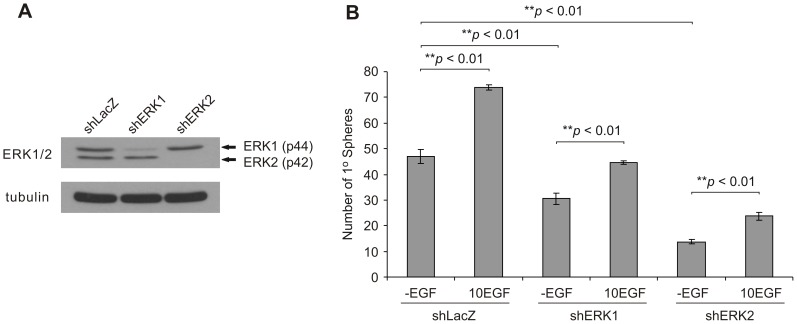
DU145 sphere formation is significantly reduced by ERK1 and ERK2 knockdown. A) Western blot analysis of endogenous ERK1 and ERK2 (ERK1/2) protein levels following ERK1 or ERK2 knockdown in DU145 monolayer cells. B) ERK1 or ERK2 knockdown results in a significant reduction in primary sphere formation compared to shRNA control (shLacZ) cells. Cells were seeded at a density of 2.5×10^3^ cells/well (0.5 ml/well; three replicates per treatment). The number of primary (1°) spheres formed in the absence (-EGF) or presence of 10 ng/ml EGF (10EGF) is displayed as mean ± S.E.M. of three independent experiments (the *p* values for the indicated comparisons were obtained by two-tailed independent Student’s *t*-test).

To further consolidate the notion that ERK signaling directly influences PCSC propagation, we have knocked-down either ERK1 or ERK2 in DU145 cells using specific shRNAs (Figure 6A). These ERK1 and ERK2 shRNA target sequences have previously been verified to efficiently knockdown either ERK1 or ERK2 in human multiple myeloma cells [19] and MCF7 breast cancer cells [38]. Knockdown of endogenous ERK1 or ERK2 was capable of significantly reducing primary sphere formation compared to shRNA control (shLacZ) cells (Figure 6B). Moreover, EGF treatment was still capable of enhancing sphere formation when either ERK1 or ERK2 was knocked-down, but not when MEK1 kinase activity was compromised (Figure 5). This suggests that redundancy in ERK1 and ERK2 function likely compensates in EGF-mediated enhancement of sphere formation when either ERK1 or ERK2 is knocked-down.

## Discussion

Cancer stem cells (CSCs) are widely regarded to play a major role in cancer progression. In order to better understand CSCs, an essential aspect of CSC research is to develop the capacity to faithfully propagate CSCs. The established system in this domain is to culture CSCs as spheres under SF media conditions supplemented with specific growth factors (GFs) [39]. In terms of PCSC propagation, the commonly used GFs are EGF and bFGF. While the combination of both of these GFs was capable of generating spheres from DU145 cells [11], [12], [17], the presence of bFGF actually reduced EGF’s ability to promote the propagation of DU145 spheres [11]. Therefore, evidence suggests that EGFR signaling is critical in maintaining the self-renewal capacity of DU145 PCSCs.

Our research reported here further supports the critical contribution of EGFR signaling to DU145 PCSC self-renewal. While propagation of DU145 PCSCs does not require the presence of exogenous EGF, activation of EGFR signaling occurred in this condition and more importantly, the activation of EGFR was also functionally important. This was based on the observation that inhibition of EGFR signaling reduced DU145 sphere cell propagation in the absence of exogenous EGF (Figures 3 and 4). Despite the mechanism leading to the activation of EGFR signaling without the addition of exogenous EGF requires further investigation, multiple possibilities can be envisaged, including the production of EGF or other ligands competent in activating EGFR in an autocrine fashion, as well as ligand-independent activation of EGFR. In our effort to determine whether autocrine-derived EGF-like ligands are responsible for the activation of EGFR in PCSCs, we examined the effect of treating sphere cells with an EGFR blocking antibody [40]. This antibody has been used to reduce EGFR-mediated self-renewal in human brain tumor stem cells [21]. While this antibody reduced EGF-enhanced formation of DU145 spheres, the antibody did not effect DU145 sphere formation under the -EGF media condition (Figure S3). In conjunction with the observation that the EGFR inhibitor AG1478 potently inhibited sphere formation (Figure 3B), evidence suggests the involvement of ligand-independent EGFR activity in DU145 sphere propagation. Although we could not exclude the possibility of autocrine production of EGF or other EGFR-activating ligands (i.e. TGF-α), it is tempting to suggest that ligand-independent activation of EGFR may contribute towards EGFR activation in the -EGF media condition. Mutations in EGFR resulting in its ligand-independent activation were reported in prostate cancer. For instance, the EGFRvIII mutant [41] and a number of EGFR tyrosine kinase domain mutants [42] that display activated EGFR signaling have been detected in prostate cancer. These mutants enhanced EGFR signaling, cell proliferation and transforming ability in the absence of EGF stimulation [42]. However, future research is needed to identify the mechanisms responsible for EGFR activation in DU145 PCSCs under the -EGF media condition.

Our finding that EGFR signaling is critical for DU145 PCSCs is consistent with the emergence of results that support EGFR in playing an essential role in CSCs. Although EGFR is a prognostic marker of various advanced stage carcinomas such as breast [43], ovarian [44], prostate [45], [46] and non-small cell lung cancers [47], the role of EGFR expression and signaling in CSCs has only recently been investigated. Enhanced levels of EGFR expression have been associated with the stem cell population in prostate cancer tissue [48]. In human brain cancer, exogenous EGF significantly enhances the formation of neurosphere cultures, while inhibition of EGFR tyrosine kinase activity potently inhibits sphere formation [49]. EGFR expression identifies tumor-initiating cells with different tumorigenic capacities in human glioblastoma multiforme [50]; enforced EGFR expression in CSCs enhanced their tumorigenic potential, while ectopic EGFR expression increased the in vivo tumorigenic capacity of EGFR-negative CSC lines. In addition, EGFR knockdown in EGFR^+^ CSCs decreased their tumorigenicity in vivo and promoted differentiation of these cells. Furthermore, there is evidence suggesting that EGFR-high expressing cells display stem cell characteristics [50].

While downstream of EGFR lies the PI3K-AKT, MEK-ERK and STAT3 pathways, we define here that the MEK-ERK pathway contributes to EGFR’s role in maintaining the “stemness” of DU145 PCSCs. This is consistent with the inhibition of self-renewal observed following MEK inhibition in human glioblastoma cancer stem-like cells [51] and human breast tumor-initiating cells [52]. While MEK-ERK activation is necessary and sufficient to mediate Raf-induced androgen receptor (AR) downregulation in prostate cancer cell lines irrespective of AR sensitivity [53], patients who have failed hormone ablation therapy display elevated ERK1/2 signal activation in recurrent tumors [54]. However, we like to stress that our research does not exclude the possibility that the PI3K-AKT pathway also contributes to the self-renewal of DU145 PCSCs.

## Supporting Information

Figure S1 **EGF treatment of DU145 PCSCs promotes EGFR signal and downstream MAPK (ERK) signal activation.** Low passage DU145 spheres, which were cultured and maintained in EGF-free serum-free media containing 0.4% BSA and 0.2× B27 (-EGF), were treated with serum-free media containing 10 ng/ml EGF (10EGF) and whole cell lysates were prepared at different time points (hours; h) post-treatment. Western blot analysis of EGF treatment of spheres results in EGFR (Tyr1068 phosphorylation; EGFR-P), ERK (Thr202/Tyr204 phosphorylation; ERK1/2-P) and STAT3 (Tyr705 phosphorylation; STAT3-P) signal activation. For each sample, a total of 100 µg of whole cell lysate was used.
(TIF)

Figure S2 **U0126 treatment reduces ERK activation in DU145 PCSCs.** Western blot analysis of whole cell lysates (50 µg of lysate was used for each sample) following 24 hour treatment of DU145 spheres with a 50 µM dose of U0126 (MEK inhibitor) or dimethylsulfoxide (DMSO; mock treatment) at an equal volume. ERK activation was determined by examining the phosphorylation of ERK1 and ERK2 proteins at Thr202 and Tyr204 residues, respectively (ERK1/2-P).
(TIF)

Figure S3 **Treatment of DU145 PCSCs with an EGFR function blocking antibody inhibits EGF-enhanced sphere formation.** Individualized cells from EGF-free DU145 spheres were treated (at the time of seeding) with azide-free anti-EGFR blocking mouse monoclonal antibody, or mouse IgG2 isotype control antibody, at a concentration of 4 µg/ml. Sphere cells were seeded at a density of 2×10^3^ cells/well (0.5 ml/well; three replicates per treatment) in serum-free media lacking EGF (-EGF) or containing 10 ng/ml EGF (10EGF). The number of spheres that formed was counted 12 days post-seeding. Sphere numbers are displayed as mean ± S.E.M. (*p<0.05; two-tailed independent Student’s *t*-test).
(TIF)
