# Supplementary figures and images for: Propagation of Human Prostate Cancer Stem-Like Cells Occurs through EGFR-Mediated ERK Activation
Source: PLoS One. 2013 Apr 19;8(4):e61716. doi: 10.1371/journal.pone.0061716 (PMC3631151; doi:10.1371/journal.pone.0061716)

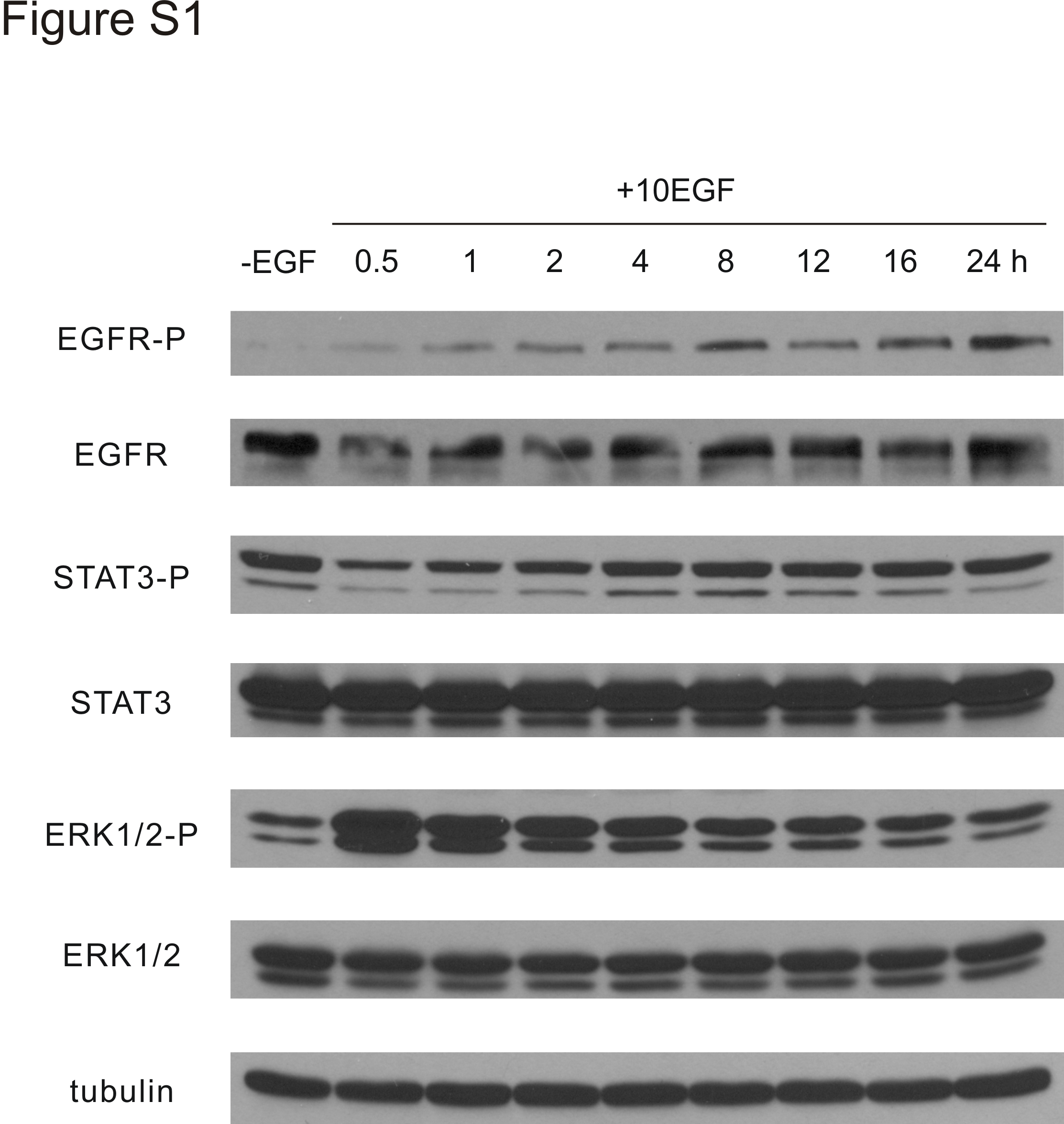

Supplement: Figure S1 — EGF treatment of DU145 PCSCs promotes EGFR signal and downstream MAPK (ERK) signal activation. Low passage DU145 spheres, which were cultured and maintained in EGF-free serum-free media containing 0.4% BSA and 0.2× B27 (-EGF), were treated with serum-free media containing 10 ng/ml EGF (10EGF) and whole cell lysates were prepared at different time points (hours; h) post-treatment. Western blot analysis of EGF treatment of spheres results in EGFR (Tyr1068 phosphorylation; EGFR-P), ERK (Thr202/Tyr204 phosphorylation; ERK1/2-P) and STAT3 (Tyr705 phosphorylation; STAT3-P) signal activation. For each sample, a total of 100 µg of whole cell lysate was used. (TIF) [file pone.0061716.s001.tif]

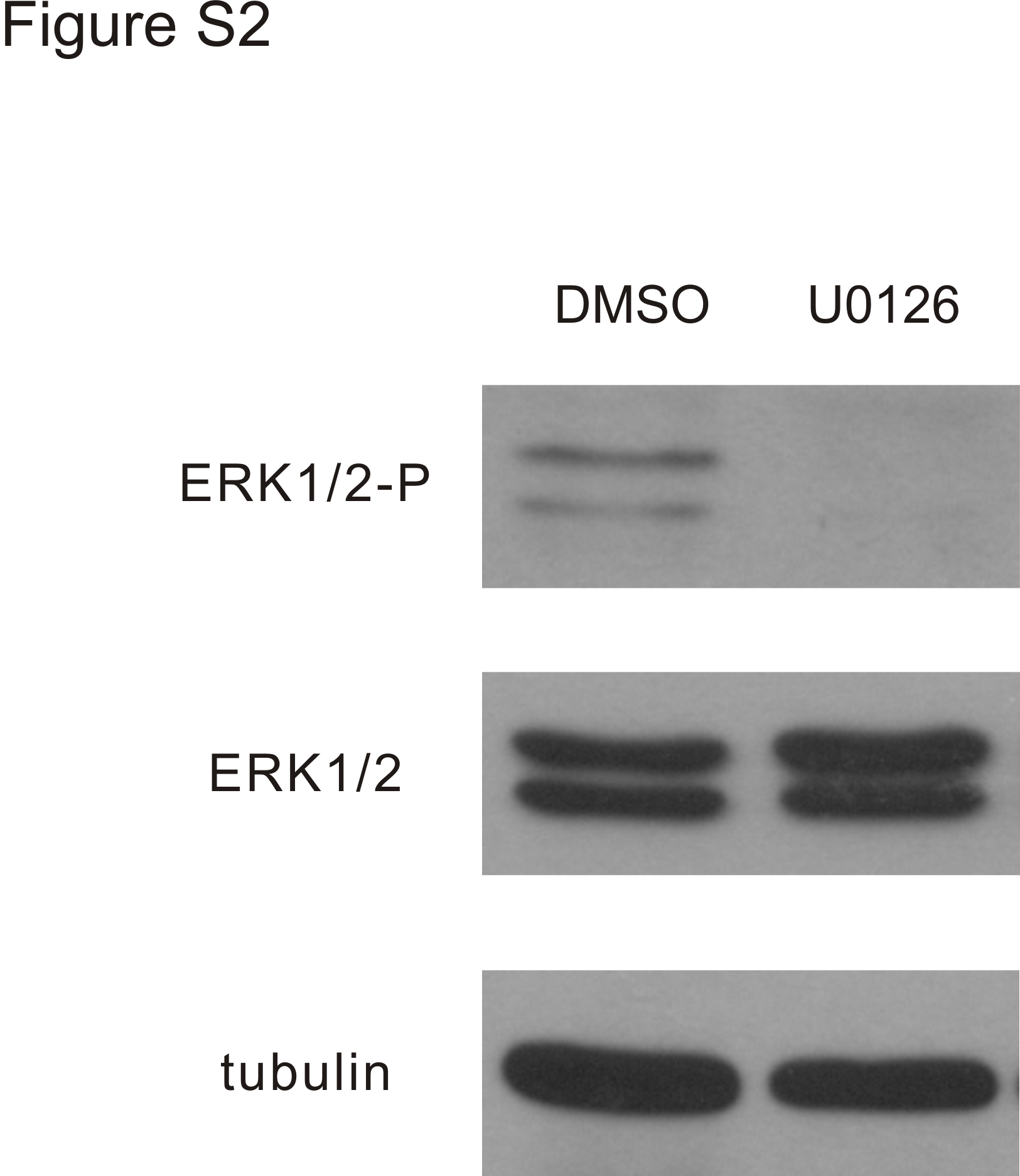

Supplement: Figure S2 — U0126 treatment reduces ERK activation in DU145 PCSCs. Western blot analysis of whole cell lysates (50 µg of lysate was used for each sample) following 24 hour treatment of DU145 spheres with a 50 µM dose of U0126 (MEK inhibitor) or dimethylsulfoxide (DMSO; mock treatment) at an equal volume. ERK activation was determined by examining the phosphorylation of ERK1 and ERK2 proteins at Thr202 and Tyr204 residues, respectively (ERK1/2-P). (TIF) [file pone.0061716.s002.tif]

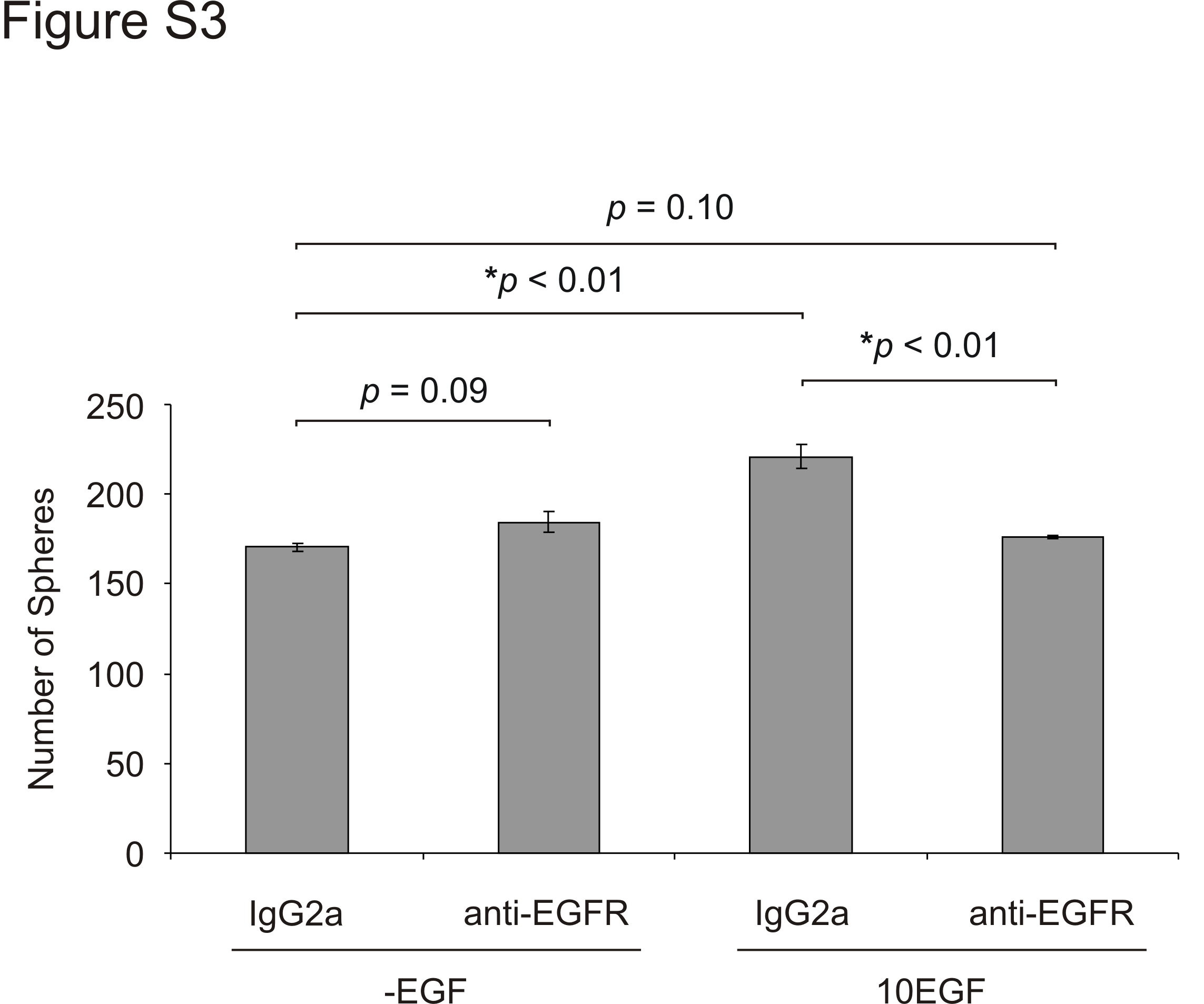

Supplement: Figure S3 — Treatment of DU145 PCSCs with an EGFR function blocking antibody inhibits EGF-enhanced sphere formation. Individualized cells from EGF-free DU145 spheres were treated (at the time of seeding) with azide-free anti-EGFR blocking mouse monoclonal antibody, or mouse IgG2 isotype control antibody, at a concentration of 4 µg/ml. Sphere cells were seeded at a density of 2×10^3^ cells/well (0.5 ml/well; three replicates per treatment) in serum-free media lacking EGF (-EGF) or containing 10 ng/ml EGF (10EGF). The number of spheres that formed was counted 12 days post-seeding. Sphere numbers are displayed as mean ± S.E.M. (*p<0.05; two-tailed independent Student’s t-test). (TIF) [file pone.0061716.s003.tif]
